# Supplementary figures and images for: The diagnostic accuracy of the Mini-Cog screening tool for the detection of cognitive impairment—A systematic review and meta-analysis
Source: PLoS One. 2024 Mar 14;19(3):e0298686. doi: 10.1371/journal.pone.0298686 (PMC10939258; doi:10.1371/journal.pone.0298686)

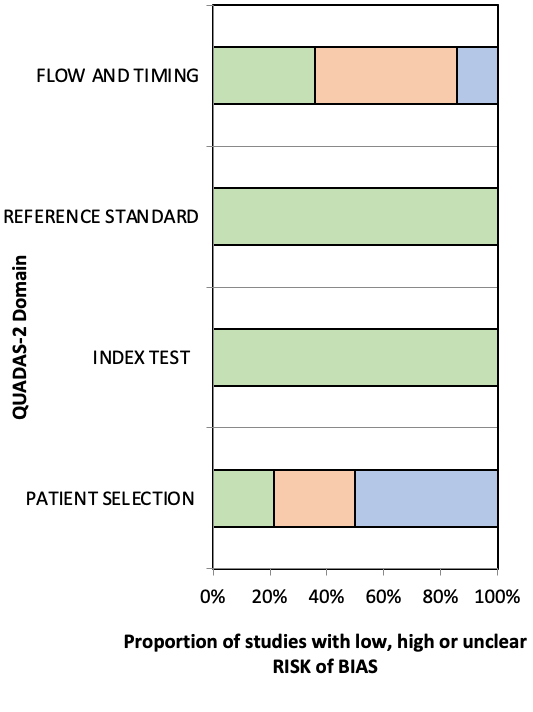

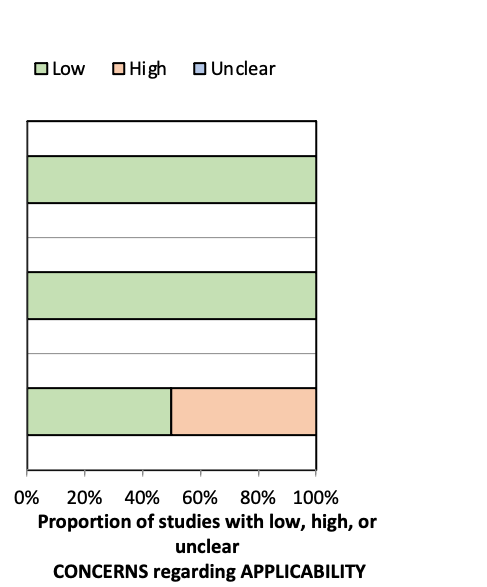


**S1 Fig. Risk of bias and applicability graphs for QUADAS-2**

Supplement: S1 Fig — (DOCX) [file pone.0298686.s001.docx]
