# Supplementary material for: The diagnostic accuracy of the Mini-Cog screening tool for the detection of cognitive impairment—A systematic review and meta-analysis
Source: PLoS One. 2024 Mar 14;19(3):e0298686. doi: 10.1371/journal.pone.0298686 (PMC10939258; doi:10.1371/journal.pone.0298686)
Supplement: S2 Fig — (DOCX) [file pone.0298686.s002.docx]

**
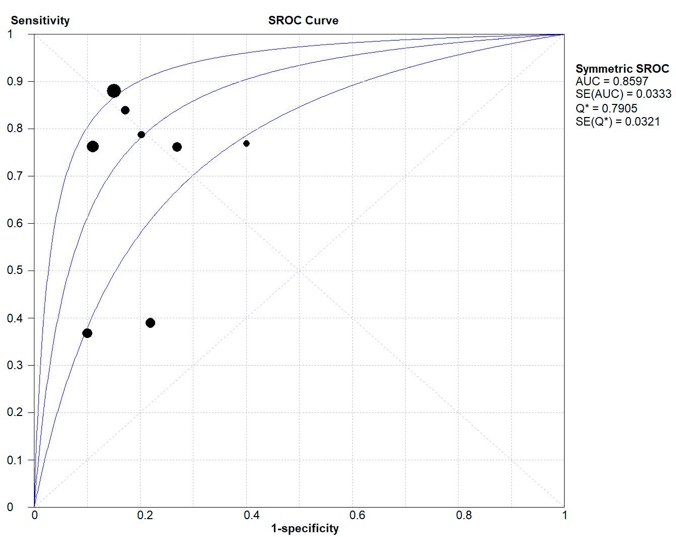

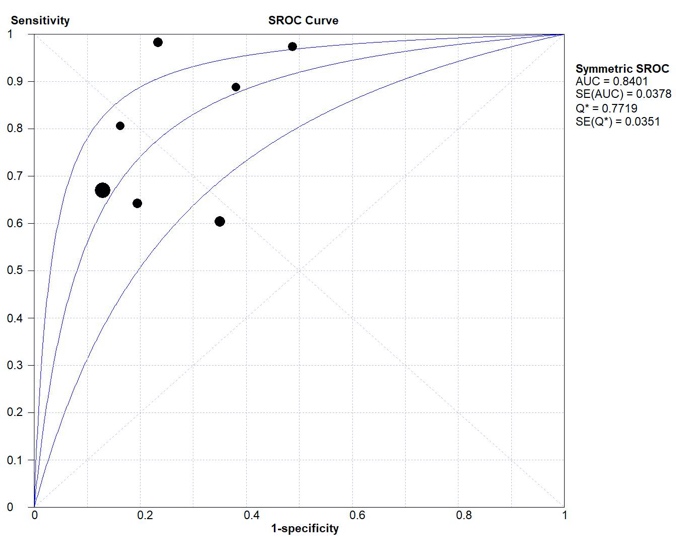
**

a) Primary care/ community b) Secondary care/ memory clinic


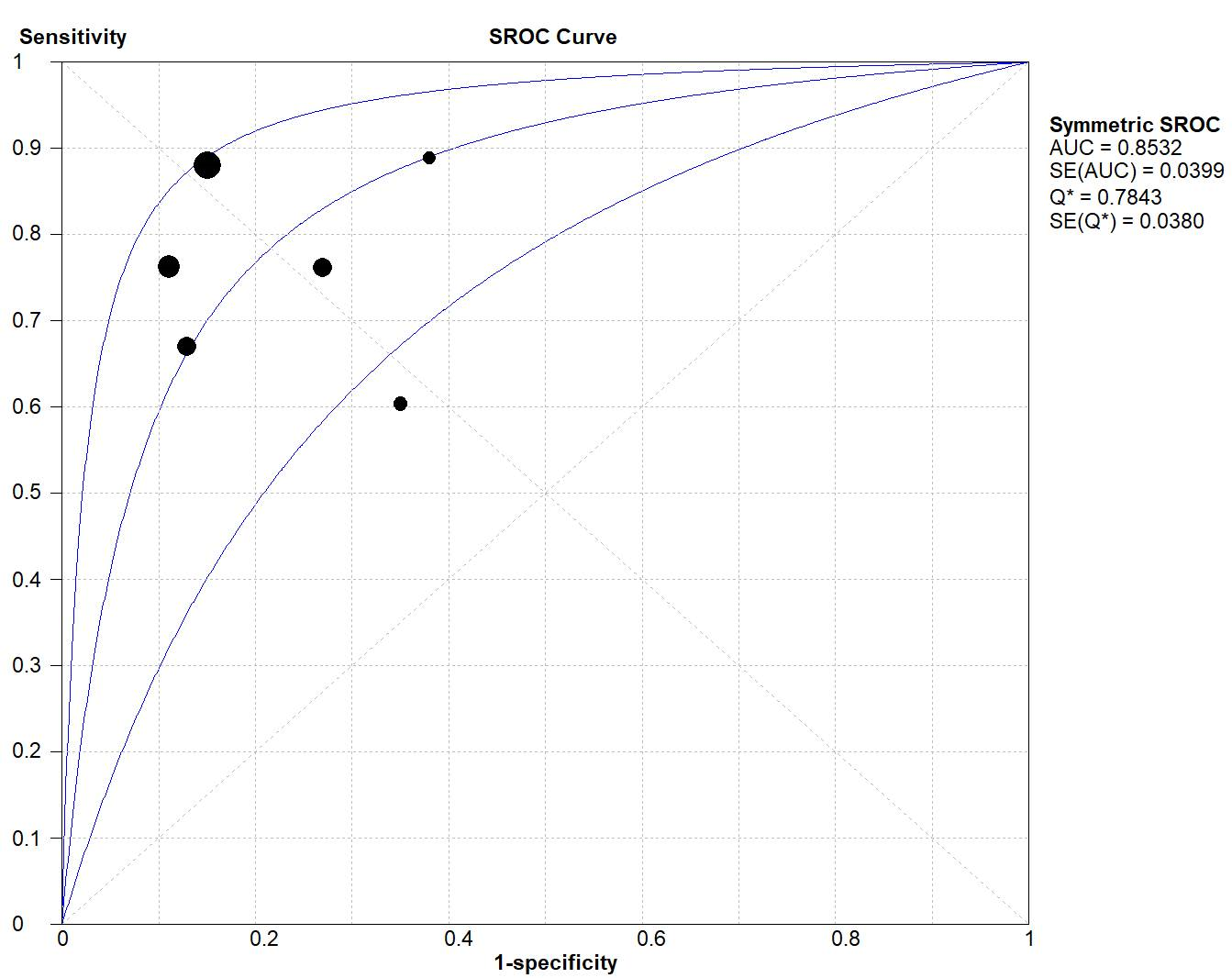

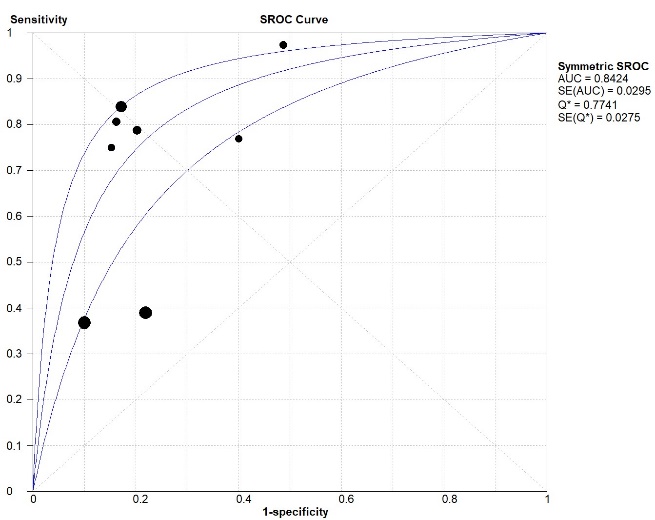


c) Dementia d) CI

Abbreviations: AUC, area under the curve; CI, cognitive impairment; Q*, point of indifference on the ROC curve; SE, standard error; SROC, summary receiver operating characteristic.

**S2 Fig. Summary receiver operating characteristic (SROC) of the Mini-Cog**
