# Supplementary material for: The diagnostic accuracy of the Mini-Cog screening tool for the detection of cognitive impairment—A systematic review and meta-analysis
Source: PLoS One. 2024 Mar 14;19(3):e0298686. doi: 10.1371/journal.pone.0298686 (PMC10939258; doi:10.1371/journal.pone.0298686)
Supplement: S1 Table — (DOCX) [file pone.0298686.s003.docx]

**S1 Table. Search Strategy**

| Databases (Platforms) | **Database Dates covered** | **Date Database  was searched** | **# Citations** | **Notes/Comments** |
| --- | --- | --- | --- | --- |
| MEDLINE (Ovid) | 1946 – January 24, 2023 | January 25, 2023 | 200 |  |
| MEDLINE ePub Ahead of Print / MEDLINE In-Process & Other Non-Indexed Citations (Ovid) | 2023 January 24 | January 25, 2023 | 62 | No limits available |
| Embase (Ovid) | 1947 – January 24, 2023 | January 25, 2023 | 264 | Conference and non-journal materials removed at source |
| Cochrane Central Register of Controlled Trials (Ovid) | 1991 – December 2022 | January 25, 2023 | 15 | Conference and non-journal materials removed at source |
| Cochrane Database of Systematic Reviews (Ovid) | 2005 – January 18, 2023 | January 25, 2023 | 16 |  |
| APA PsycINFO (Ovid) | 1806 – Jan. Week 3 2023 | January 25, 2023 | 184 | Conference and non-journal materials removed at source |
| Web of Science Core Collection (Clarivate Analytics) | 1900 – Jan. 23, 2023 | January 25, 2023 | 343 | Conference and non-journal materials removed at source |
| Scopus (Elsevier) | 1960 – present | January 25, 2023 | 331 | Conference and non-journal materials removed at source |
|  |  | Totals: | 1415 | Results in RIS formatted files |
| **Citing Citation Searching** |  |  |  |  |
| **Citing †Borson 2000** |  |  |  |  |
| Scopus (Elsevier) | 1960 – present | January 25, 2023 | 940 | Conference and non-journal materials removed at source |
| Web of Science Core Collection (Clarivate Analytics) | 1900 – Jan. 23, 2023 | January 25, 2023 | 879 | Conference and non-journal materials removed at source |
| *Citation Chaser (Lens.Org) | n/a | January 25, 2023 | 1031 | **NB: 189 citations were removed from the original 1220 citations obtained by Citation Chaser (non-journal) |
|  |  |  | 2850 |  |

**†The mini-cog: a cognitive 'vital signs' measure for dementia screening in multi-lingual elderly,** S. Borson, J. M. Scanlan, M. Brush, P. P. Vitaliano and A. Dokmak, International journal of geriatric psychiatry 2000 Vol. 15 Issue 11 Pages 1021-1027, DOI: 10.1002/1099-1166(200011)15:11<1021::aid-gps234>3.0.co;2-6

NB: citing the use of the Citation Chaser:

*Haddaway, N. R., Grainger, M. J., Gray, C. T. (2021) citationchaser: An R package and Shiny app for forward and backward citations chasing in academic searching. doi: [10.5281/zenodo.4543513](https://www.doi.org/10.5281/zenodo.4543513)

**Search Strategies Used**

**The mini-cog: a cognitive 'vital signs' measure for dementia screening in multi-lingual elderly**

S. Borson, J. M. Scanlan, M. Brush, P. P. Vitaliano and A. Dokmak

International journal of geriatric psychiatry 2000 Vol. 15 Issue 11 Pages 1021-1027

DOI: 10.1002/1099-1166(200011)15:11<1021::aid-gps234>3.0.co;2-6

# MEDLINE

Ovid MEDLINE(R) 1946 to January 24, 2023

| # | Searches | Results |
| --- | --- | --- |
| 1 | "mini-cog??".mp. | 204 |
| 2 | minicog??.mp. | 7 |
| 3 | 1 or 2 | 210 |
| 4 | remove duplicates from 3 | 209 |
| 5 | limit 4 to english language | 200 |

# MEDLINE In-Process

Ovid MEDLINE(R) Epub Ahead of Print and In-Process, In-Data-Review & Other Non-Indexed Citations January 24, 2023

| # | Searches | Results |
| --- | --- | --- |
| 1 | "mini-cog??".mp. | 62 |
| 2 | minicog??.mp. | 2 |
| 3 | 1 or 2 | 64 |
| 4 | remove duplicates from 3 | 63 |
| 5 | limit 4 to english language | 62 |

# Embase

Embase Classic+Embase 1947 to 2023 January 24

| # | Searches | Results |
| --- | --- | --- |
| 1 | "mini-cog??".mp. | 598 |
| 2 | minicog??.mp. | 71 |
| 3 | 1 or 2 | 652 |
| 4 | remove duplicates from 3 | 627 |
| 5 | limit 4 to english language | 612 |
| 6 | limit 5 to (conference abstracts or "preprints (unpublished, non-peer reviewed)" or (books or chapter or conference abstract or conference paper or "conference review" or "preprint (unpublished, non-peer reviewed)") or (book or book series or conference proceeding or "preprint archive (unpublished, non-peer reviewed)" or trade journal)) | 348 |
| 7 | 5 not 6 [ Removal of Conference and non-journal material ] | 264 |

# CCTR

EBM Reviews - Cochrane Central Register of Controlled Trials December 2022

| # | Searches | Results |
| --- | --- | --- |
| 1 | "mini-cog??".mp. | 63 |
| 2 | minicog??.mp. | 8 |
| 3 | 1 or 2 | 69 |
| 4 | remove duplicates from 3 | 68 |
| 5 | limit 4 to english language | 67 |
| 6 | 5 not (abstract or addresses or bibliography or biography or book or book article or book book or book note or "book review" or book series article or book series article in press or book series chapter or book series conference paper or book series letter or "book series review" or book series short survey or chapter or conference or conference abstract or conference abstract placebo controlled partly blinded crossover study in 12 sle patients or conference proceeding or "conference review" or journal conference abstract or "journal conference review" or monograph or conferenc* or book*).pt. | 51 |
| 7 | 6 not Trial registry record*.pt. [ Removal of empty trial records ] | 15 |
| 8 | 6 not 7 [ double-check ] | 36 |
| 9 | 7 [ Final Results ] | 15 |

# CDSR

EBM Reviews - Cochrane Database of Systematic Reviews 2005 to January 18, 2023

| # | Searches | Results |
| --- | --- | --- |
| 1 | "mini-cog??".mp. | 10 |
| 2 | minicog??.mp. | 7 |
| 3 | 1 or 2 | 16 |
| 4 | remove duplicates from 3 | 16 |

# APA PsycINFO

APA PsycInfo 1806 to January Week 3 2023

| # | Searches | Results |
| --- | --- | --- |
| 1 | "mini-cog??".mp. | 225 |
| 2 | minicog??.mp. | 10 |
| 3 | minicog.tm. | 6 |
| 4 | mini-cog.tm. | 183 |
| 5 | or/1-4 | 234 |
| 6 | remove duplicates from 5 | 234 |
| 7 | limit 6 to english language | 231 |
| 8 | limit 7 to ("0100 journal" or "0110 peer-reviewed journal" or "0120 non-peer-reviewed journal" or "0130 peer-reviewed status unknown") | 184 |
| 9 | limit 7 to ("0200 book" or "0240 authored book" or "0280 edited book" or "0300 encyclopedia" or "0400 dissertation abstract" or (classic book or conference proceedings or "handbook/manual" or reference book or "textbook/study guide")) | 47 |
| 10 | 7 not 9 [ double-check ] | 184 |
| 11 | 8 or 10 | 184 |

# Web of Science

Web of Science Search Strategy (v0.1)

Database: Web of Science Core Collection

Entitlements:

- WOS.SCI: 1900 to 2023

- WOS.AHCI: 1975 to 2023

- WOS.BHCI: 2005 to 2023

- WOS.BSCI: 2005 to 2023

- WOS.ESCI: 2005 to 2023

- WOS.ISTP: 1990 to 2023

- WOS.SSCI: 1900 to 2023

- WOS.ISSHP: 1990 to 2023

Mini-Cog or MiniCog English Jrls only

Data updated 2023-01-23

# Searches:

Search: "mini-cog##" OR "minicog##" (All Fields) and English (Languages) and Article or Review Article or Early Access or Proceeding Paper or Letter (Document Types)

Date Run: Wed Jan 25 2023 09:26:04 GMT-0500 (Eastern Standard Time) Results: 343

Search: "mini-cog##" OR "minicog##" (All Fields) and English (Languages)

Date Run: Wed Jan 25 2023 09:25:28 GMT-0500 (Eastern Standard Time) Results: 385

Search: "mini-cog##" OR "minicog##" (All Fields)

Date Run: Wed Jan 25 2023 09:25:06 GMT-0500 (Eastern Standard Time) Results: 400

Search: "mini-cog#" OR "minicog#" (All Fields)

Date Run: Wed Jan 25 2023 09:24:52 GMT-0500 (Eastern Standard Time) Results: 400

Search: "mini-cog*" OR "minicog*" (All Fields)

Date Run: Wed Jan 25 2023 09:23:48 GMT-0500 (Eastern Standard Time) Results: 427

# Scopus

Mini-Cog or MiniCog English Jrls only

**331 document results**

TITLE-ABS-KEY ( "mini-cog*"  OR  "minicog*" )  AND  ( LIMIT-TO ( LANGUAGE ,  "English" ) )  AND  ( LIMIT-TO ( DOCTYPE ,  "ar" )  OR  LIMIT-TO ( DOCTYPE ,  "re" )  OR  LIMIT-TO ( DOCTYPE ,  "cp" )  OR  LIMIT-TO ( DOCTYPE ,  "le" ) )

CITATION SEARCHING OF:

Borson S, Scanlan J, Brush M, Vitaliano P, Dokmak A.

The mini-cog: a cognitive 'vital signs' measure for dementia screening in multi-lingual elderly.

Int J Geriatr Psychiatry. 2000 Nov;15(11):1021-7. doi: 10.1002/1099-1166(200011)15:11<1021::aid-gps234>3.0.co;2-6.

PMID: 11113982.

- - 1. Web of Science Core Collection
    2. Scopus
    3. *CitationChaser

NB: citing the use of the Citation Chaser:

*Haddaway, N. R., Grainger, M. J., Gray, C. T. (2021) citationchaser: An R package and Shiny app for forward and backward
