# Supplementary material for: The diagnostic accuracy of the Mini-Cog screening tool for the detection of cognitive impairment—A systematic review and meta-analysis
Source: PLoS One. 2024 Mar 14;19(3):e0298686. doi: 10.1371/journal.pone.0298686 (PMC10939258; doi:10.1371/journal.pone.0298686)
Supplement: S2 Table — (DOCX) [file pone.0298686.s004.docx]

**S2 Table. QUADAS-2: Quality Assessment of Diagnostic Accuracy Studies**

|  | **PATIENT SELECTION** | **INDEX TEST** | **REFERENCE STANDARD** | **FLOW AND TIMING** |
| --- | --- | --- | --- | --- |
| Borson 2003 | **UNC** | **Low** | **Low** | **High** |
| Borson 2005 | UNC | Low | Low | UNC |
| Costa 2012 | UNC | Low | Low | UNC |
| Clionsky 2010 | UNC | Low | Low | Low |
| Filho 2009 | High | Low | Low | High |
| Limpawattan 2021 | High | Low | Low | Low |
| Holsinger 2012 | Low | Low | Low | High |
| Kemenski 2009 | Low | Low | Low | High |
| Ketelaars 2013 | UNC | Low | Low | Low |
| Pourshams 2022 | High | Low | Low | Low |
| Razaei 2018 | High | Low | Low | High |
| Shang 2021 | UNC | Low | Low | Low |
| Wilber 2015 | UNC | Low | Low | High |
| Yang 2016 | Low | Low | Low | High |

Applicability table:

|  | **PATIENT SELECTION** | **INDEX TEST** | **REFERENCE STANDARD** |
| --- | --- | --- | --- |
| Borson 2003 | **Low** | **Low** | **Low** |
| Borson 2005 | High | Low | Low |
| Costa 2012 | Low | Low | Low |
| Clionsky 2010 | High | Low | Low |
| Filho 2009 | High | Low | Low |
| Limpawattan 2021 | High | Low | Low |
| Holsinger 2012 | Low | Low | Low |
| Kemenski 2009 | Low | Low | Low |
| Ketelaars 2013 | High | Low | Low |
| Pourshams 2022 | Low | Low | Low |
| Razaei 2018 | High | Low | Low |
| Shang 2021 | High | Low | Low |
| Wilber 2015 | Low | Low | Low |
| Yang 2016 | Low | Low | Low |
