# Supplementary material for: The diagnostic accuracy of the Mini-Cog screening tool for the detection of cognitive impairment—A systematic review and meta-analysis
Source: PLoS One. 2024 Mar 14;19(3):e0298686. doi: 10.1371/journal.pone.0298686 (PMC10939258; doi:10.1371/journal.pone.0298686)
Supplement: S3 Table — (DOCX) [file pone.0298686.s005.docx]

**S3 Table. Predictive Parameters of included studies using Mini-Cog to detect dementia, mild cognitive impairment (MCI) and cognitive impairment (CI)**

| Study | Sensitivity | Specificity | PPV | NPV | AUROC | PLR | NLR |
| --- | --- | --- | --- | --- | --- | --- | --- |
| *Community/Primary Care* | | | | | | | |
| Borson 2003 | 0.76 | 0.89 | 0.34 | 0.98 | NR | 6.91 | 0.27 |
| Borson 2005 | 0.84 | 0.83 | 0.89 | 0.76 | NR | 4.94 | 0.19 |
| Costa 2012 | 0.37 | 0.9 | 0.01 | 0.98 | NR | 3.51 | 0.71 |
| Holsinger 2012 | 0.76 | 0.73 | 0.08 | 0.99 | 0.84 | 2.81 | 0.33 |
| Holsinger 2012 | 0.39 | 0.78 | 0.57 | 0.63 | 0.64 | 1.76 | 0.79 |
| Kemenski 2009 | 0.77 | 0.6 | 0.45 | 0.86 | NR | 1.93 | 0.38 |
| Shang 2021 | 0.79 | 0.80 | 0.73 | 0.84 | 0.79 | 3.90 | 0.27 |
| Yang 2016 | 0.88 | 0.85 | 0.63 | 0.96 | 0.87 | 5.96 | 0.15 |
| *Secondary care/memory clinic* | | | | | | | |
| Clionksy 2010 | 0.67 | 0.87 | 0.94 | 0.49 | 0.84 | 5.15 | 0.38 |
| Filho 2009 | 0.6 | 0.65 | 0.45 | 0.77 | 0.676 | 1.71 | 0.62 |
| Ketelaars 2013 | 0.81 | 0.84 | 0.68 | 0.91 | NR | 4.98 | 0.23 |
| Ketelaars 2013 | 0.97 | 0.51 | 0.8 | 0.91 | NR | 2.00 | 0.05 |
| Limpawattan 2021 | 0.64 | 0.81 | 0.57 | 0.85 | 0.73 | 3.92 | 0.5 |
| Pourshams 2022 | 0.98 | 0.77 | 0.98 | 0.81 | 0.94 | 4.26 | 0.03 |
| Rezaei 2018 | 0.88 | 0.63 | 0.68 | 0.86 | 0.71 | 2.37 | 019 |
| *Emergency Department* |  |  |  |  |  |  |  |
| Wilber 2015 | 0.75 | 0.85 | 0.57 | 0.93 | NR | 5.00 | 0.29 |
