# Supplementary material for: The diagnostic accuracy of the Mini-Cog screening tool for the detection of cognitive impairment—A systematic review and meta-analysis
Source: PLoS One. 2024 Mar 14;19(3):e0298686. doi: 10.1371/journal.pone.0298686 (PMC10939258; doi:10.1371/journal.pone.0298686)
Supplement: Attachment — Submitted filename: Comments to the authors.docx [file pone.0298686.s007.docx]

**Comments to the authors:**

This systematic review and meta-analysis evaluated the diagnostic accuracy of the Mini-Cog tool to screen for cognitive impairment (CI) across various healthcare settings, mainly primary and secondary care settings. A qualitative analysis including the QUADAS-2 criteria for risk of bias assessment and an evidence table were used, and for quantitative analyses, the random-effects bivariate analysis of the included studies was conducted. The authors found that the sensitivity and specificity of the Mini-Cog to detect either MCI, dementia, or CI in primary care settings were 73% and 84%, respectively, whereas those of the secondary care settings were 73% and 76%, respectively. High and unclear risk of bias were found for patient selection and time intervals.

The merit of this study is the use of proper statistical methods for meta-analysis and a comprehensive qualitative analysis; however, this is highly hindered by the unclear definition and report of cognitive impairment based on the Mini-Cog score, which impedes the clinical feasibility and interpretation of the findings. Please kindly find below my points of concern that need to be addressed and some recommendations.

**Overall:**

L37-39, L61-62: The authors reported that the cut-off of ≤2/5 (according to the scoring rules published b Borson et. Al., 2000) was used to classify dementia, MCI, and CI (defined as either MCI or dementia)”; however, the authors are encouraged to report the cut-points used to distinguish dementia from MCI, especially, when they reported studies where MCI was detected (L45); otherwise, grouping MCI and Dementia as CI is quite misleading. Clarifying this will help not only enhance the potential value of this manuscript but also the applicability of the findings by helping clinicians make a more informed clinical decision.

For this reason, the authors are encouraged to redefine their purpose and definition of terms, improve the presentation of results and keep consistency of the report of the findings.

**Introduction:**

- L96-100: The authors indicate that previous reviews have reported the accuracy of the Mini-Cog to detect only dementia and imply that the diagnostic accuracy of the Mini-Cog to detect MCI or CI (either MCI or dementia). Please reconsider redefining your purpose or definition of term as commented above.

**Results:**

- PRISMA flow diagram: Please add the number of records identified from each database.
- Line 181 and Table 2: Although the authors reported that 14 studies were included for the final analysis, 15 studies are mentioned in Table 2, and Figures 2 and 3, and 16 studies are mentioned in Table 1. I assume this is because the authors reported two separate data from the same study, but this should be clearly reported in the Results section.

**Discussion:**

- Please report the findings in the first paragraph of this section.
- Table 2, for the Secondary care/memory clinic, the prevalence is higher than that of the primary/community care (0.58 vs 0.20), but also the specificity of the Mini-Cog test is lower than the primary/community care (0.76 vs 0.84). Do these values affect the prevalence in each healthcare setting? Please, kindly discuss these differences.

**Tables and Figures:**

- To ease readability, kindly add the reference number for each study in Tables 1 and 2 and Figures 2 and 3, just like it was reported in the main text (L193-194).
- Table 1: Please describe the Mini-Cog score used to define Dementia and MCI. As much as possible, I encourage authors to check the studies where they reported CI. For instance, Table 1 of the study of Borson et al., 2005 shows a classification of CI into “Dementia Probably AD,” “Vascular Dementia,” “Other Dementia,” and “MCI.”
- Figures 2 and 3: Please include the AUC values.
- Table 2: Please clearly indicate in the description that the values of the Healthcare settings are for CI in general; otherwise, please indicate the cognitive status reported in the studies for each healthcare setting.

**Minor points:**

- L135: A semicolon is needed between “(AUC)” and “5)”.
- L262: "setting" should be "settings"
- L202-203 and Table 2 of the Primary/community care, the AUC values are different. This also applies to the AUC and sensitivity values of the Secondary care/memory clinic.
- L213-217 and Table 2 of the Emergency department: Although in the main text, the authors report one study in the emergency department, in Table 2, this is reported as "NA". Please confirm.
