# Supplementary material for: The diagnostic accuracy of the Mini-Cog screening tool for the detection of cognitive impairment—A systematic review and meta-analysis
Source: PLoS One. 2024 Mar 14;19(3):e0298686. doi: 10.1371/journal.pone.0298686 (PMC10939258; doi:10.1371/journal.pone.0298686)
Supplement: Attachment — Submitted filename: Response to Reviewers.doc [file pone.0298686.s008.doc]

**Reply to the reviewers’ comments**

**Thank you for these insightful comments. We would like to clarify that we have repeated one meta-analysis as we added a missing paper which was brought to our attention by a reviewer's comment. Specifically, the Kemenski et al. 2009 paper was added to the cognitive impairment subgroup. Minor changes to the predictive parameters have also been reflected throughout the paper.**

**After moving one supplementary table into the main text as suggested by the reviewers, we have relabelled the order of our supplementary materials.**

| Reviewer Number | Original comments of the reviewer | Reply by the author(s) | Changes done on page number and line number |
| --- | --- | --- | --- |
| 1 | The authors are encouraged to report the cut-points used to distinguish dementia from MCI, especially, when they reported studies where MCI was detected (L45); otherwise, grouping MCI and Dementia as CI is quite misleading. Clarifying this will help not only enhance the potential value of this manuscript but also the applicability of the findings by helping clinicians make a more informed clinical decision. | Our study focuses on determining the accuracy of the Mini-Cog to detect cognitive impairment (CI), which according to the DSM 5 includes both mild CI (MCI) and major CI (dementia). The original creator of the Mini-Cog, Borson, first developed the Mini-Cog to detect dementia (2000). He applied the Mini-Cog to detect varying severities of cognitive impairment (MCI and dementia) using the original cutoff (Borson 2005).  Several studies have utilized the Mini-Cog and other screening tools to detect CI which have been described as both MCI and dementia in their methodology (Tanwani 2023, Holsinger 2012, Shang 2021). To date, there has been no systematic review and meta-analysis identifying the accuracy of the Mini-Cog to detect CI in different health care settings. Given this, we wanted to utilize the same cutoff in our inclusion criteria to have consistency with the established literature and to determine if this existing cutoff may be appropriately applied to screen for CI across different severities, including MCI and dementia for different health care settings. We have clarified this in our introduction (L98-106).  We have added that the same cutoff was used across all settings (L44). | L 44  L98-106 |
| 1 | L96-100: The authors indicate that previous reviews have reported the accuracy of the Mini-Cog to detect only dementia and imply that the diagnostic accuracy of the Mini-Cog to detect MCI or CI (either MCI or dementia). Please reconsider redefining your purpose or definition of term as commented above. | We have clarified our terms within this section with the specification that we aim to determine the accuracy of the Mini-Cog across varying severities of cognitive impairment which includes MCI and dementia in different populations and various health care settings. | L103-104 |
| 1 | PRISMA flow diagram: Please add the number of records identified from each database. | Revised accordingly | Fig 1. |
| 1 | Line 181 and Table 2: Although the authors reported that 14 studies were included for the final analysis, 15 studies are mentioned in Table 2, and Figures 2 and 3, and 16 studies are mentioned in Table 1. I assume this is because the authors reported two separate data from the same study, but this should be clearly reported in the Results section. | Thanks for raising this point. One study was left out of the analysis within Table 2 which has now been added and all values have been updated. The total number of articles included were 14. Two sets of data were used from two different studies.  We have clarified that two separate datasets from two studies were included in our study L194-195. In total, there were 16 datasets. | L194-195  Table 2 |
| 1 | Please report the findings in the first paragraph of this section (Discussion). | Revised accordingly | Line 277-283 |
| 1 | Table 2, for the Secondary care/memory clinic, the prevalence is higher than that of the primary/community care (0.58 vs 0.20), but also the specificity of the Mini-Cog test is lower than the primary/community care (0.76 vs 0.84). Do these values affect the prevalence in each healthcare setting? Please, kindly discuss these differences. | Thank you for raising this point. We have discussed these differences in our discussion. | Page 16 L 297-303 |
| 1 | To ease readability, kindly add the reference number for each study in Tables 1 and 2 and Figures 2 and 3, just like it was reported in the main text (L193-194). | Revised accordingly. | Table 1 and 2  Figure 2 and 3 |
| 1 | Table 1: Please describe the Mini-Cog score used to define Dementia and MCI. As much as possible, I encourage authors to check the studies where they reported CI. For instance, Table 1 of the study of Borson et al., 2005 shows a classification of CI into “Dementia Probably AD,” “Vascular Dementia,” “Other Dementia,” and “MCI.” | In our methodology, specifically the statistics section, we discussed that a cut-off of two or less was used as the threshold for having dementia, MCI, or CI (defined as either dementia or MCI). Within Borson 2005, the original author of the Mini-Cog used this same cut off, ≤2/5 for Dementia, Vascular Dementia and MCI. We have added further clarification as suggested within the table’s caption. | Table 1 |
| 1 | Figures 2 and 3: Please include the AUC values. | Revised accordingly | Fig 2 and 3 |
| 1 | Table 2: Please clearly indicate in the description that the values of the Healthcare settings are for CI in general; otherwise, please indicate the cognitive status reported in the studies for each healthcare setting. | Revised accordingly | Table 2 |
| 1 | L135: A semicolon is needed between “(AUC)” and “5)”. | Revised accordingly. | Page 6, line 140 |
| 1 | L262: "setting" should be "settings" | Revised accordingly. | Page 16, lines 285 |
| 1 | L202-203 and Table 2 of the Primary/community care, the AUC values are different. This also applies to the AUC and sensitivity values of the Secondary care/memory clinic. | Revised accordingly. | Line 210, 219 |
| 1 | L213-217 and Table 2 of the Emergency department: Although in the main text, the authors report one study in the emergency department, in Table 2, this is reported as "NA". Please confirm. | Revised accordingly. There is only one study in the emergency dept. The predictive parameters were added into Table 2. | Table 2 |
| 2 | Please clarify whether the Mini-Cog was initially developed to screen dementia and mild cognitive impairment. If my knowledge is correct, the cognitive test was developed for screening dementia. Please make clearer and justify further the theoretical rationale behind the extension beyond the original intended use. | The Mini-Cog was initially published to screen for dementia, which has now been clarified on Line 92. The use of the Mini-Cog to screen for MCI has become increasingly popular and there is an increasing need to screen for MCI within clinical settings. We have discussed this with further clarification in our introduction, (L100-106) and discussion (315-320). | Page 5 Line 100-106  Page 18 Line 315-320. |
| 2 | Results: If possible, it would be better to conduct publication bias test for diagnostic accuracy of the Mini-Cog by funnel plot analysis. | While we understand the importance of publication bias, we are unable to complete the funnel plot analysis as there are less than 10 studies within each subgroup. |  |
| 2 | Discussion: In this study, two diagnostic accuracy measures (i.e., sensitivity and specificity) were analyzed. Considering the inherent trade-off between sensitivity and specificity, it might be better to add AUC as the outcome measure. As Table 2 shows, however, the pooled AUC was not calculated in studies to detect mild cognitive impairment. At least, this issue should be acknowledged, as well as the small number of studies, in the Limitations subsection on page 18. | We have now added the AUC to our figures (Fig 2, Fig 3) to further emphasize its importance as an outcome measure.  Since there were only two studies on MCI, we cannot calculate a pooled AUC. This was added to the limitations (L356). | Fig 2, 3  L 356 |
| 2 | Results: If I did not overlook, please spell out abbreviations such as D and ND in Table 1. Also, the standard deviations of age seem lacking in Borson et al. (2005). | Revised accordingly. Standard deviations for this study were not provided by Borson et al 2005. | Table 1 |
| 2 | Results: Although Table 2 summarizes the pooled sensitivity and specificity, it would be better to present them in Figure 2 as well. If I understood correctly, the authors presented the sensitivity and specificity in each study only. | The pooled sensitivity and specificity as presented within the text of Figure 2 successfully presents this information. We have also added AUC into figure 2 and 3. | Figure 2 |
| 2 | Results: If I understood correctly, S1 Table shows the results of meta-regression exploring the factors of the heterogeneity between studies. However, the descriptions on page 14 seem to be about the leave-one-study-out” meta-analysis. Please check and revise the text and supplementary materials if needed. Furthermore, if allowed, it would be better to present S1 Table in the main text. | Revised text accordingly. We have also moved the S1 table into the main text (Table 3, Table 4). | Line 245-256  Table 3 Table 4 |
| 2 | Discussion: It might be better to refer to references #13 and #15 in the second paragraph in the Discussion section on page 15 and to clarify the contribution of this study. In the previous reviews, the number of studies was limited, and the evidence to recommend that the Mini-Cog should be used as a screening test for dementia was insufficient. | We have revised this paragraph to include the limitations of previous reviews. | Line 313-314 |
| 2 | Discussion: Considering the limited number of studies to detect mild cognitive impairment by the Mini-Cog and the pooled sensitivity and specificity, I had a concern that the authors’ conclusion that the Mini-Cog is comparable to the MoCA on page 17 could be overemphasized. Further examinations would be needed to test whether the diagnostic accuracy of the Mini-Cog is comparable to the MoCA. | Thanks for the suggestion. Revised accordingly. | Line 324-329 |
| 2 | Discussion: When referring to the first limitation that this study included case-control studies, it would be better to mention that the previous reviews excluded case-control studies if I understood correctly. | We wanted to highlight the inherent bias of using case control studies in systematic reviews in our study. | Page 18, Line 350 |
