# Supplementary material for: The diagnostic accuracy of the Mini-Cog screening tool for the detection of cognitive impairment—A systematic review and meta-analysis
Source: PLoS One. 2024 Mar 14;19(3):e0298686. doi: 10.1371/journal.pone.0298686 (PMC10939258; doi:10.1371/journal.pone.0298686)
Supplement: Attachment — Submitted filename: Response to Reviewers Jan 11th.doc [file pone.0298686.s009.doc]

**Reply to the reviewers’ comments**

| Reviewer Number | Original comments of the reviewer | Reply by the author(s) | Changes done on page number and line number |
| --- | --- | --- | --- |
| 1 | Line 31: From the authors’ response, “To date, there has been no systematic review and meta-analysis identifying the accuracy of the Mini-Cog to detect CI in different health care settings.”, please emphasize that the accuracy of the Mini-Cog to detect CI in different health care settings is unclear. | This has been emphasized in L33 | L 33 |
| 1 | Lines 38-39 and 44-45: The sentences are repetitive. Following the authors’ response, please clarify in L44-45 that the cutoff was used across all settings. Please, make sure which symbol is appropriate “≤” (Line 38) or “≥” (Line 44). | L 44-45 has been removed. The appropriate “≤” symbol has been confirmed on Line 39. | L39. |
| 1 | Discussion: Since the findings are being reported in the first paragraph of the Discussion section, please combine lines 280 and 281 together. | Revised accordingly | L 282,283 |
| 2 | Introduction: To address the major comment #1, the authors referred to earlier studies using the Mini-Cog to screen mild cognitive impairment. However, it would be better to clarify the theoretical, not empirical, rationale behind the extension of the scope of the Mini-Cog. Specifically, how could the authors justify that the two cognitive tests (i.e., word recall and clock drawing) assess mild cognitive impairment? | MCI involves a decline across several cognitive domains without a limitation in function. The CDT and word recall allow for the assessment of various domains including memory, visuospatial skills and execute function that are known to decline in MCI. This has been described in the discussion (L325-328) to further expand on why on the Mini-Cog may be applied to screen MCI. | L323-326 |
| 2 | Discussion: To address the minor comment #9, the authors explained the limitation of the inclusion of case-control studies in the response letter in addition to the main text. However, if I understand the previous studies correctly, it would be fair to briefly explain that previous reviews excluded case-control studies and to clarify the weakness of the current review in the Limitations subsection. If my understanding is incorrect, the authors do not have to mention the previous reviews in the subsection. | Revised accordingly | Line 356-357 |
