# Supplementary material for: The diagnostic accuracy of the Mini-Cog screening tool for the detection of cognitive impairment—A systematic review and meta-analysis
Source: PLoS One. 2024 Mar 14;19(3):e0298686. doi: 10.1371/journal.pone.0298686 (PMC10939258; doi:10.1371/journal.pone.0298686)
Supplement: Attachment — Submitted filename: Response to Reviewers Jan 23rd.doc [file pone.0298686.s010.doc]

**Reply to the reviewers’ comments**

| Reviewer Number | Original comments of the reviewer | Reply by the author(s) | Changes done on page number and line number |
| --- | --- | --- | --- |
| 1 | Line 33: Please combine the sentences together using a transition word, such as "however" and punctuate it accordingly. | This has been revised accordingly. | L 32 |
| 1 | Line 102: Please define "NCD" at first mention. | This has been revised accordingly. | L 78 |
